# In Response

**DOI:** 10.4269/ajtmh.2010.09-0646b

**Published:** 2010-03

Dear Sir:

First of all, we appreciate all comments from Dr. Tsai and others on our article. We agree that characteristics of eosinophilic meningitis caused by *Angiostrongylus cantonensis* in Thailand and Taiwan are different. In Thailand, most patients are adults and fever is less common. In contrast, most patients in Taiwan are children and fever is more common. These differences might be caused by the route of infection and carrying hosts. Adult Thai patients acquire the disease by eating raw golden apple snails (*A. canaliculatus*) or other paratenic hosts; whereas exposure to the African giant snails/slugs (*A*. *fulica*) is the main cause of disease in Taiwanese children.^1^ In addition, adults and children may have different immune responses to the parasites. Encephalitic form is severe and causes severe neurological sequele. Our study indicates risk factors of encephalitic angiostrongyliasis only in adults. We encourage Taiwanese researchers to evaluate the risks of developing encephalitic angiostrongyliasis in children.

Regarding the exclusion criteria, we intended to exclude patients only if the computed tomography (CT) or magnetic resonance imaging (MRI) of the brain indicated other causes of cerebrospinal fluid (CSF) eosinophils, particularly gnathostomiasis. Previous reports showed some radiographic features indicating gnathostomiasis.^2^–^6^ If any patients had abnormal findings of CT or MRI of the brain compatible with angiostrongyliasis, they were not excluded. In our study, no patient was excluded because of this criterion.

We would like to thank Dr. Tsai and others for mentioning our proposed sample size calculation. We would like to clarify the calculation and add a missing reference.^7^ According to the mentioned statistical setting, the preliminary numbers of subjects in the encephalitic and meningitic groups would be 15 and 91, respectively (meningitic/encephalitic groups equal 6:1).^8^ However, we retrospectively gathered data and had limitation by 14 subjects of encephalitic angiostrongyliasis. With the number of meningitic angiostrongyliasis of 86 subjects, the power of our study will be 78% or approximately 80%.^7^ Our results showed that the proportions of subjects who had fever in meningitic and encephalitic groups were 10% and 71%, respectively. When we re-calculated by using these results, the power of our study was almost 100%.^7^

Finally, we agree that to determine the risk factors of developing encephalitic angiostrongyliasis needs further prospective observation. And, it might be different among age groups and places.

Kittisak Sawanyawisuth

E-mail: kittisak@kku.ac.th
